# Combination of dura turning-over and decompressive craniectomy: a new pattern of surgery for cerebral infarction caused by craniocerebral gunshot injury

**DOI:** 10.1186/s40779-017-0135-4

**Published:** 2017-08-17

**Authors:** Qi-Yong Mei, Yao Li, Chao He, Hong-Wei Shan, Yun-Kun Wang, Yan Dong, Ming-Kun Yu, Li-Jun Hou

**Affiliations:** 1 Department of Neurosurgery, Shanghai Changzheng Hospital, the Second Military Medical University, Shanghai Neurosurgical Institute, Shanghai, 200003 China; 2 Department of Hepatic Surgery III, Eastern Hepatobiliary Surgical Hospital, Shanghai, 200438 China; 3 Department of Emergency, Shanghai Changzheng Hospital, the Second Military Medical University, Shanghai, 200003 China

**Keywords:** Gunshot cerebral injury, Infarction, Dura turning-over, Decompressive craniectomy

## Abstract

**Background:**

Craniocerebral gunshot injury refers to a wound caused by a bullet passing through or lodged in brain tissue, resulting in the loss of function of a certain area or other fatal damage to the human brain. Craniocerebral gunshot injury is usually life-threatening and is very common in modern warfare, accounting for the majority of battle casualties. Most of the patients suffer from acute cerebral infarction caused by vascular injury. Lack of early and solid battlefield emergency medical interference adds to the risk of death among the wounded.

**Case presentation:**

We present a 24-year-old man who was shot with a shotgun from a distance of 15 m in an accidental injury. Forty-seven grapeshots were found on his body surface by physical examination. A computed tomography (CT) scan demonstrated large areas of low-density shadows in his right parietal lobe and right temporal lobe with the midline shifting to the left side 2 days later. Afterwards, the patient was transferred to our emergency medical center at Changzheng Hospital in Shanghai. Cranial computed tomography angiography (CTA) showed a high-density shadow in the initial part of the right middle cerebral artery. The branches after the initial part were obliterated. Prompt medical attention and decompressive craniotomy (DC) surgery contributed to the final recovery from cerebral infarction of this patient.

**Conclusion:**

Bullets can penetrate or be lodged in the brain, causing intracranial hypertension. The bullets lodged in the brain can result in stenosis and embolism of a cerebral artery, causing acute cerebral infarction. Combining dura turning-over surgery with DC surgery can not only decrease intracranial pressure, which can increase the blood supply for hypertension-induced vessel stenosis, but also help vessels outside the dura mater grow into ischemic areas of the cerebral cortex. However, this new pattern of surgery needs further support from evidence-based medicine.

## Background

Since the late 1950s, firearm deaths have increased dramatically in the United States. Every year, guns are responsible for more than 32,000 deaths in the country. Suicide accounted for 53% of the fatalities and 40% of homicides. Accidental shootings and those with undetermined causes made up the remainder. Gunshot injuries to the head are the most lethal of all the firearm injuries, ranking among the leading causes of head injury in many United States cities. Each year, close to 20,000 persons in the United States are involved in gunshot wounds to the head [1, 2]. They carry a fatality rate greater than 90%, and at least two-thirds of the victims die before reaching a hospital. For victims who survive the initial trauma, approximately 50% die in the emergency room. The extent of damage is dependent on a number of factors, such as the magnitude of energy transferred, distance traveled by the missile, type of bullet, and the structures encountered before and on penetration. In general, high-energy transfer gun-shots fired at close range inflict the most damage [3]. Gunshot brain injuries are much more common and life-threatening in modern war for lack of immediate examinations and early treatments.

Acute cerebral infarction after gunshot injuries is a very common complication that can be fatal and result in the loss of function of certain brain areas, which can last for a life time if it cannot be corrected in time. Early and accurate examinations and diagnosis are of significant importance to reduce the high rate of morbidity and disability. Today, the common treatments for gunshot injuries include therapies to relieve symptoms and DC or craniotomy surgery to release the intracranial hypertension. However, some research has shown that the rates of moderate disability and good recovery with surgery were similar to those with medical management [4].

## Case presentation

**Fig. 1:**
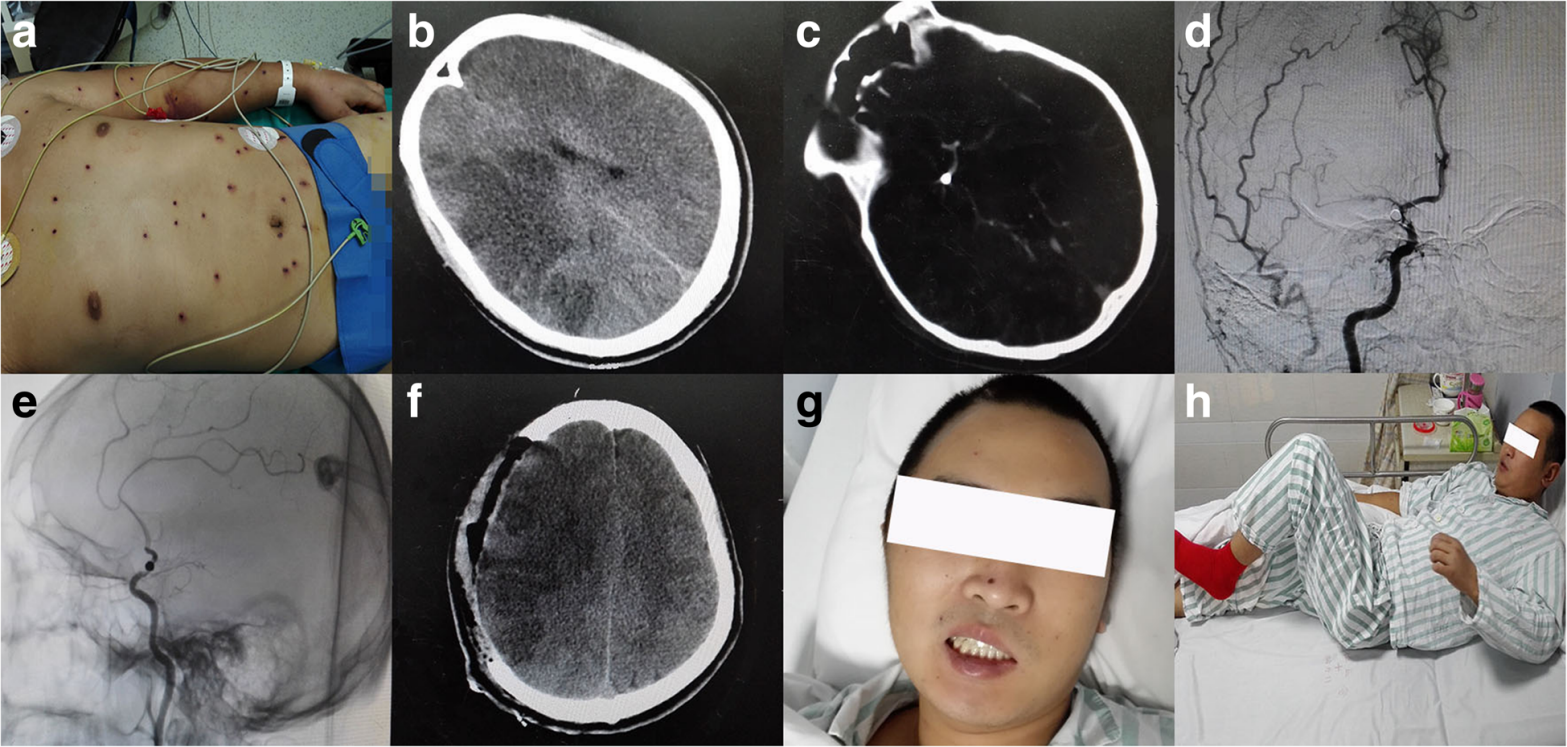
Picture and CT image of the patient. The patient with gunshot wounds showed recovery of the intracranial blood supply and physical ability gradually due to our contribution of surgery.**a** Multiple gunshot wounds in the skin of this patient; **b** CT scan revealing large parts of low-density shadows in the right frontal lobe, right parietal lobe and right temporal lobe before the surgery; **c** CT scan revealing the pellet lodged in the brain; **d, e** DSA showed the branches after the initial part of the right middle cerebral artery (MCA) were obliterated; **f** CT scan showed decreased low-density areas with the midline moving back 24 h after the surgery; **g** Posterior-anterior picture of the patient 2 weeks after the surgery showed a decreased difference between two sides of the face; **h** The patient left the hospital with myodynamia degree III of his left upper limb, degree V of the proximal part of his left lower limb and degree V of the distal part of his left lower limb

A 24-year-old man suffered from an accidental gunshot injury on December 15, 2015 (Fig. 1a). An emergency CT scan showed pellets in the saddle area of the brain (Fig. 1c), pericardium, stomach and intestines. On evaluation, he was in stable condition with a normal sinus rhythm on electrocardiography and normal blood pressure. Symptoms such as nausea, vomiting, dizziness, dystaxia, hyperspasmia and reduction in myodynamia were not found. The patient received fluid infusion, dehydration, analgesia and some other therapies to relieve his symptoms.

Alalia, facial paralysis of the left side, reduction in myodynamia of left upper and lower limbs emerged the next day. A CT scan was performed immediately that revealed large areas of infarction in his right parietal lobe and right temporal lobe. Mannitol was used to relieve his symptoms but did not take a favorable turn. The patient was admitted to Changzheng Hospital in Shanghai on December 17, 2015. Physical examination of the nervous system showed myodynamia degree II of his left upper limb and degree III of his left lower limb, reduction in the left biceps reflex and triceps reflex, reduction in the left knee reflex and Achilles’ tendon reflex. Hoffmann’s sign, Babinski’s sign and Kernig’s sign were all negative.

Cranial CTA and digital subtraction angiography (DSA) was performed immediately and it showed a high-density shadow in the initial part of the right middle cerebral artery (Fig. 1d, e). The branches after the initial part were obliterated. The right anterior and right posterior cerebral artery compensated for the blood supply of the right middle cerebral artery. However, a CT scan showed large areas of low-density shadows in the right parietal lobe and right temporal lobe with midline shifting to the left side 2 days later. Physical examination of the nervous system showed a myodynamia degree 0 of his left upper limb, degree I of the proximal part of his left lower limb and degree II of the distal part of his left lower limb. The patient presented with somnolence and dysphoria. He could still speak logically. His pupils were 3 mm in diameter and were of equal size with sensitive responses to light reflexes. The patient’s symptoms gradually became more serious. The patient could not talk logically and appeared irritable in the afternoon.

Although the formation of compensatory circulation had taken place, the ischemic situation of the patient’s brain tissue had not improved effectively according to the low-density areas in the CT scan examination (Fig. 1b). The patient was diagnosed as having an acute cerebral infarction. Acute cerebral infarction is a contradiction for cranial artery by-pass operations. It has been reported that DC surgery can lower intracranial pressure but there is little evidence to show that this surgery can help improve the blood supply.

**Fig. 2:**
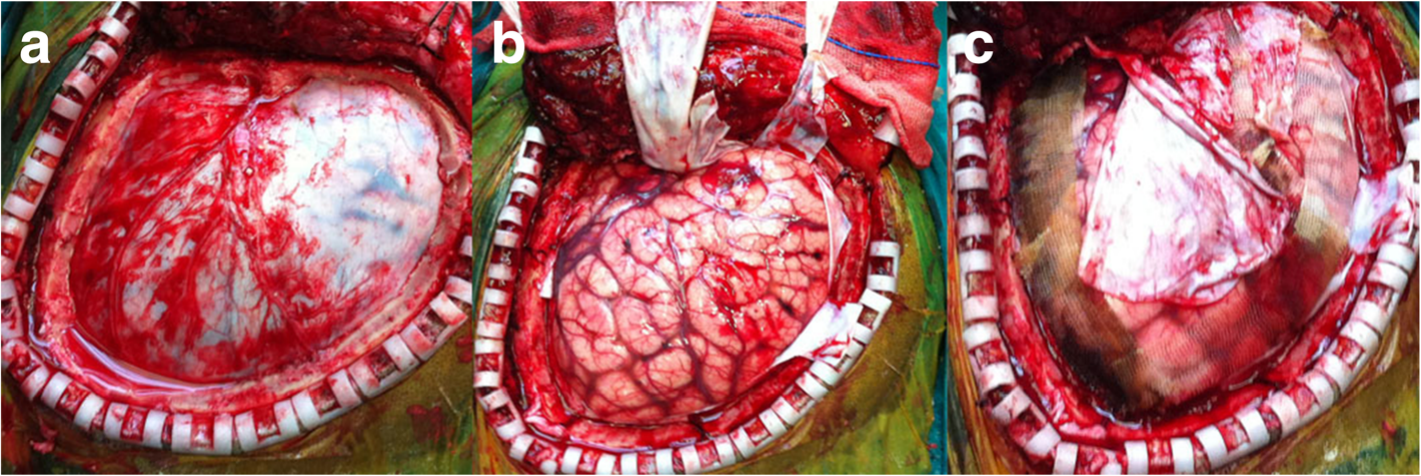
Three steps of the surgery procedure.**a** We found the area in his dura mater that we decided to turn over, while we did not cut the initial part of the vessel; **b** We turned-over this part of dura mater at the end of the initial part of the vessel; **c** A relaxed suture was used to close the incision

In terms of the dangerous and urgent situation of this patient, he was taken into the operating room. A DC and dura turning-over surgery was conducted to help him from becoming worse under general anesthesia. We removed a part of the skull in the area of the frontal bone, temporal bone and parietal bone (Fig. 2a). We made an incision in his dura mater to release the intracranial hypertension and covered his brain tissue with turning-over of his dura mater onto the brain with the outside surface (Fig. 2b, c). Afterwards, the patient’s brain artery beat rhythmically again. The incision was closed with a relaxation suture. Treatments after the surgery included hemostasis, antibiotics, analgesia, diuretics and neurotrophic drugs. CT scan showed decreased low-density areas with the midline-line moving back 24h after the surgery (Fig. 1f).

Five days after the surgery, the patient started to speak logically but was still in lethargy. Medical examination of the nervous system showed myodynamia degree I of his left upper limb, degree II of the proximal part of his left lower limb and degree II of the distal part of his left lower limb. Nine days after the surgery, the myodynamia degree of his left upper limb rose to degree II. Twelve days after the surgery, the patient turned became conscious without somnolence. Posterior-anterior picture of the patient 2 weeks after the surgery showed a decreased difference between two sides of the face (Fig. 1g). Fifteen days after the surgery, the myodynamia degree of his left lower limb rose to degree IV. Eighteen days after the surgery, medical examination of the nervous system showed myodynamia degree IV of his left upper limb, degree V of the proximal part of his left lower limb and degree V of the distal part of his left lower limb (Fig. 1h). We advised him to take more exercise to help functional recovery of his muscle and ankles. Twenty-three days after the surgery, the patient was conscious and spoke logically. Cranioplasty surgery was performed successfully 3 months later.

## Discussion

Craniocerebral penetrating gunshot injuries have a high potential to kill as wounds in action, even when treated aggressively and timely in the battlefield. The projectile will transmit the kinetic energy to the skull, fragmenting and fracturing bones, severing the brain parenchyma and generating secondary missiles that damage brain structures further [5–7].

DC is widely used for wounds in wartime. It has been reported that patients who undergo DC had worse injuries than those receiving craniotomy and, while not achieving the same outcomes as those with a lesser injury, they did improve with time. Hemicraniectomy is recommended for damage control to protect patients from the effects of brain swelling during the long overseas transport to their definitive care [3, 4]. Warfare-related DC defects can be safely reconstructed using custom alloplastic implants. The patient in our case suffered from gunshot-induced cerebral vascular injury. The feature of our case exists in the innovative pattern of surgery by the combination of dura turning-over surgery with DC surgery. Patients with craniocerebral gunshot injury should be given consideration regarding gunshot-induced vascular injury. We need to take craniocerebral vascular injury into consideration when patients fall into unconsciousness and limb dysfunction that cannot be explained by simple trauma independently.

A CT scan in time is very necessary, because it can detect foreign bodies inside cranial tissue and the secondary injury condition. CTA has remarkable superiority in regard to the battlefield condition because it can reveal the position relationship between bullets and vessels and whether the continuity of the vessel has been blocked. However, DSA is irreplaceable in the assessment of the compensatory state of the blood supply of craniocerebral vessels in the ischemic tissue.

Acute cerebral infarction is a contradiction for cranial artery by-pass operation. DC is effective in lowering intracranial pressure but there is not enough evidence to demonstrate that the surgery can improve blood supply for the ischemic tissue. There is no evidence to support the routine use of secondary DC to reduce unfavorable outcomes in adults with severe traumatic brain injury (TBI) and refractory high intracranial pressure (ICP). To date, there are no results from randomized trials to confirm or refute the effectiveness of DC in adults. However, the results of non-randomized trials and controlled trials with historical controls involving adults suggest that DC may be a useful option when maximal medical treatment has failed to control ICP [8, 9]. A study demonstrated that DC can be performed safely to lower refractory elevated ICP in pediatric TBI. Their results support the conclusion that early aggressive treatment of refractory ICP can lead to a favorable clinical status and function [10, 11].

There are abundant vessels on the surface of the dura mater. Thus, we used a new way of surgery by turning over the dura mater above the ischemic brain tissue to increase the attachment of vessels to the cerebral cortex, which often lack blood supply in a brain infarction [3, 12, 13]. Dura turning-over surgery introduced in our case can increase the blood supply and improve the ischemic state of the cerebral cortex by the growth of new vessels. Compared with an artery by-pass operation, dura turning-over surgery can be less harmful to brain tissue, which is a great advantage in protecting brain tissue from secondary injury. This new pattern of surgery is also more accurate and can improve the brain blood supply more precisely for certain brain areas. In this particular case, we successfully helped the patient recover from the edge of death. Prompt treatments and surgery all contributed to reducing neurological loss of the wounded.

A follow-up survey carried out later showed that this new pattern of surgery is effective in improving the blood supply and relieving symptoms and signs of damage to certain brain tissue. Combining dura turning-over surgery with DC can not only lower ICP, which can increase the blood supply for hypertension-induced vessel stenosis but also help vessels outside the dura mater to grow into ischemic areas of the cerebral cortex [3, 14–16]. However, it is still difficult for us to evaluate the metabolic condition of brain tissue in battlefield circumstances. Our new pattern of surgery still lacks support from evidence-based medicine. The operation should be conducted by experienced surgeons to restrain the scale of effective health care service for the army. This operation should not be conducted in emergency treatment before the base hospital within the medical treatment in echelons to ensure the safety and success rate of the surgery. The effectiveness of this surgery needs to be tested and verified by large sample and multicenter clinical research. We hope that our innovation of the surgery pattern can provide a foundation for further clinical trials to help patients with acute cerebral infarction caused by gunshot injury to receive a better prognosis in military health service support.

## Conclusion

Combination of dura turning-over and decompressive craniectomy can be a safe and efficacious option in the treatment of cerebral infarction caused by gunshot injury on the battlefield.

## Abbrevations

CT: Computed tomography
CTA: Cranial computed tomography angiography
DC: Decompressive craniectomy
DSA: Digital subtraction angiography
ICP: Intracranial pressure
TBI: Traumatic brain injury

## References

[1] Saito N, Hito R, Burke PA, Sakai O (2014). Imaging of penetrating injuries of the head and neck:current practice at a level I trauma center in the United States. Keio J Med.

[2] Bizhan A, Mossop C, Aarabi JA (2015). Surgical management of civilian gunshot wounds to the head. Handb Clin Neurol.

[3] Ongom PA, Kijjambu SC, Jombwe J (2014). Atypical gunshot injury to the right side of the face with the bullet lodged in the carotid sheath: a case report. J Med Case Rep.

[4] Bell RS, Mossop CM, Dirks MS, Stephens FL, Mulligan L, Ecker R (2010). Early decompressive craniectomy for severe penetrating and closed head injury during wartime. Neurosurg Focus.

[5] Stefanopoulos PK, Hadjigeorgiou GF, Filippakis K, Gyftokostas D (2014). Gunshot wounds: a review of ballistics related to penetrating trauma. J Acute Dis.

[6] Molina DK, Rulon JJ, Wallace EI (2012). The atypical entrance wound, differential diagnosis and discussion of an unusual cause. Am J Forensic Med Pathol.

[7] Rich NM, Johnson EV, Dimond FC (1967). Wounding power of missiles used in the republic of Vietnam. JAMA.

[8] Taylor A, Butt W, Rosenfeld J, Shann F, Ditchfield M, Lewis E (2001). A randomized trial of very early decompressive craniectomy in children with traumatic brain injury and sustained intracranial hypertension. Childs Nerv Syst.

[9] Polin RS, Shaffrey ME, Bogaev CA, Tisdale N, Germanson T, Bocchicchio B (1997). Decompressive bifrontal craniectomy in the treatment of severe refractory posttraumatic cerebral edema. Neurosurgery.

[10] Rutigliano D, Egnor MR, Priebe CJ, McCormack JE, Strong N, Scriven RJ (2006). Decompressive craniectomy in pediatric patients with traumatic brain injury with intractable elevated intracranial pressure. J Pediatr Surg.

[11] Cho DY, Wang YC, Chi CS (1995). Decompressive craniotomy for acute shaken/impact baby syndrome. Pediatr Neurosurg.

[12] Kjellberg RN, Prieto A (1971). Bifrontal decompressive craniotomy for massive cerebral edema. J Neurosurg.

[13] Rengachary SS, Batnitzky S, Morantz RA, Arjunan K, Jeffries B (1981). Hemicraniectomy for acute massive cerebral infarction. Neurosurgery.

[14] Venes JL, Collins WF (1975). Bifrontal decompressive craniectomy in the management of head trauma. J Neurosurg.

[15] Schneck MJ, Origitano TC (2006). Hemicraniectomy and durotomy for malignant middle cerebral artery infarction. Neurol Clin.

[16] Robertson SC, Lennarson P, Hasan DM, Traynelis VC (2004). Clinical course and surgical management of massive cerebral infarction. Neurosurgery.

